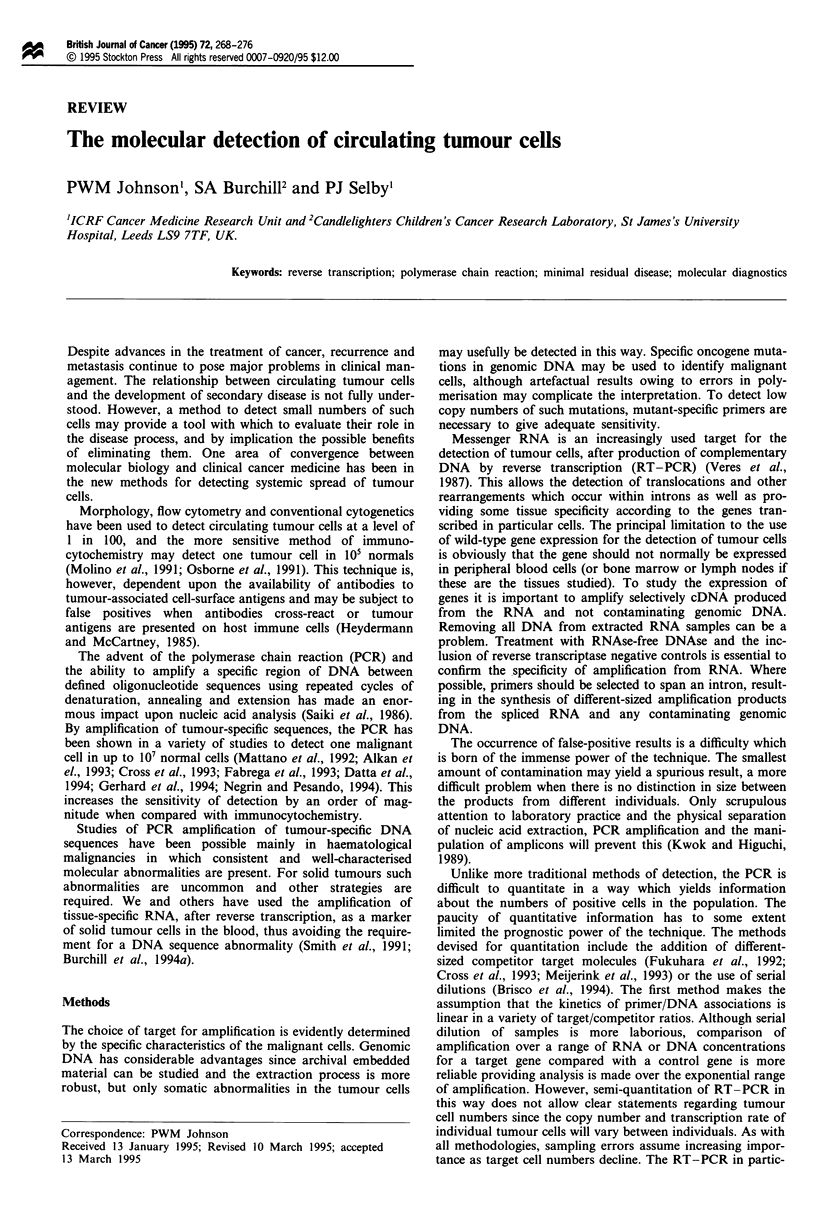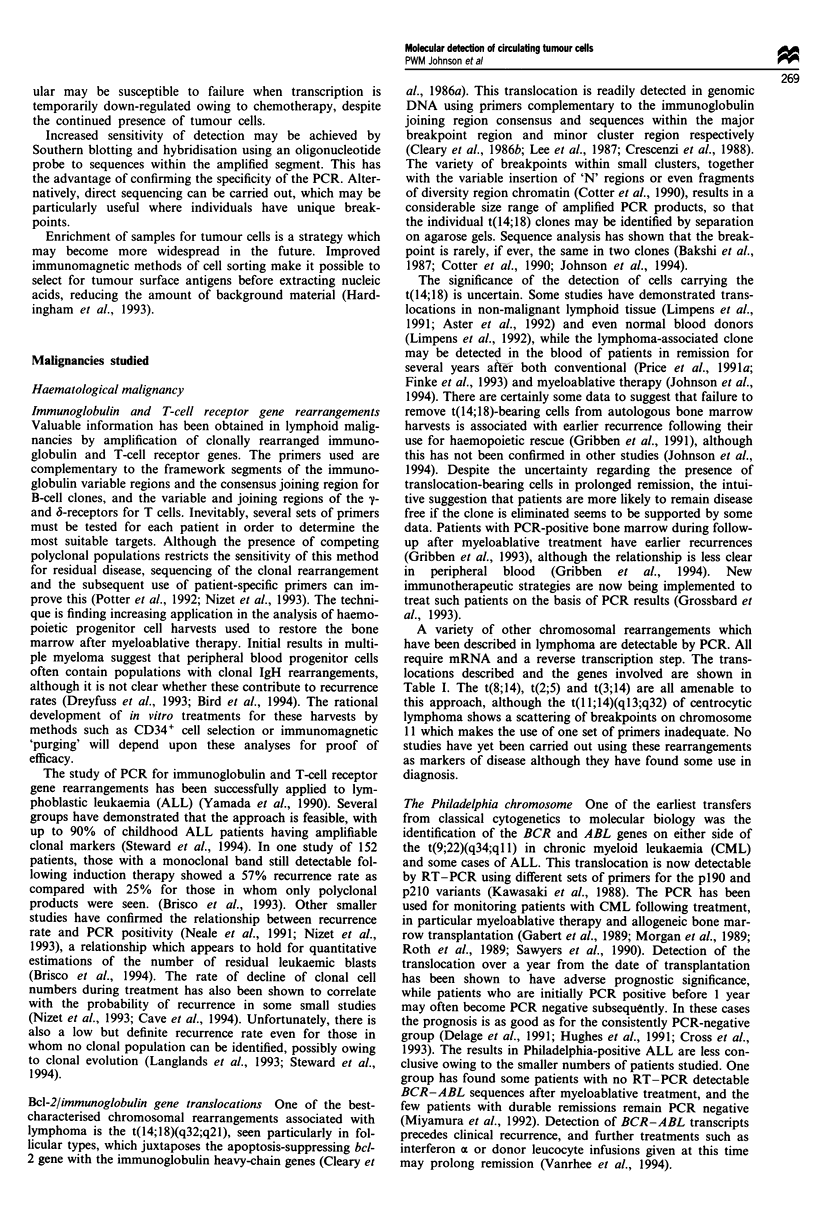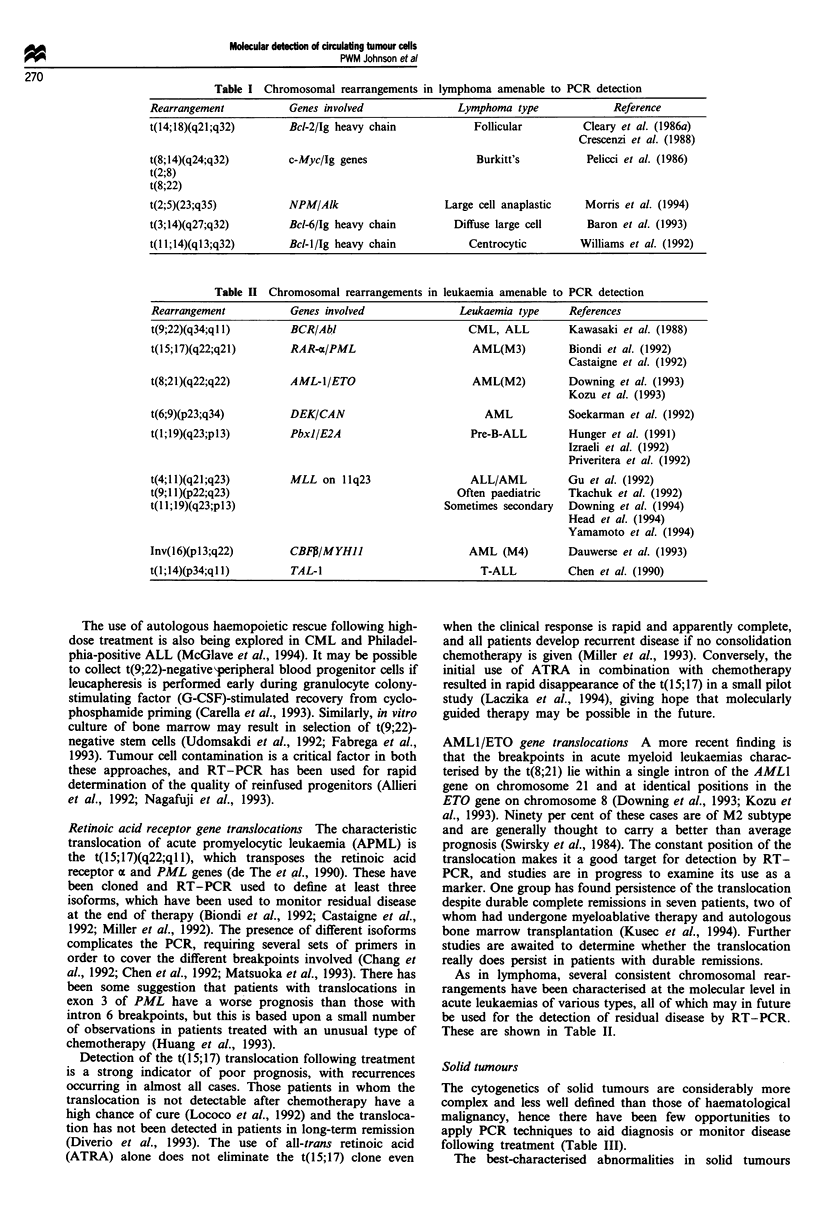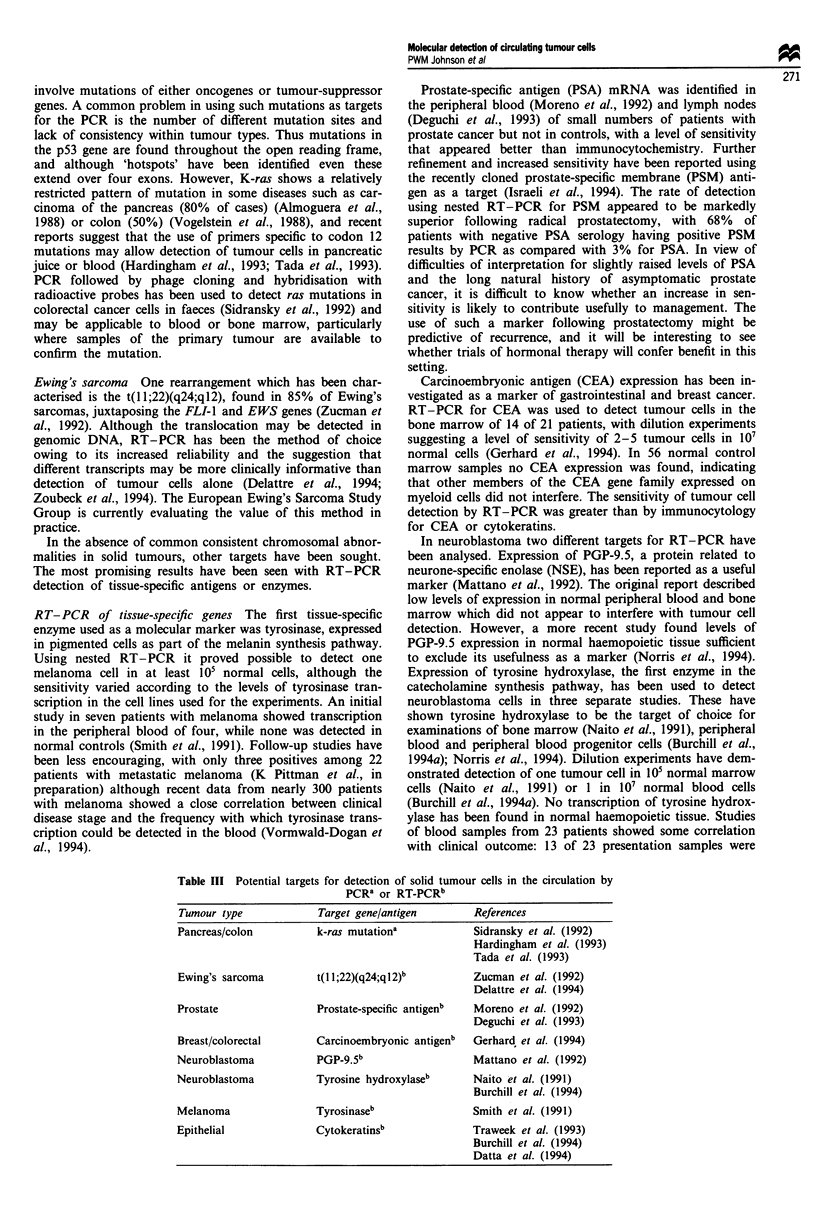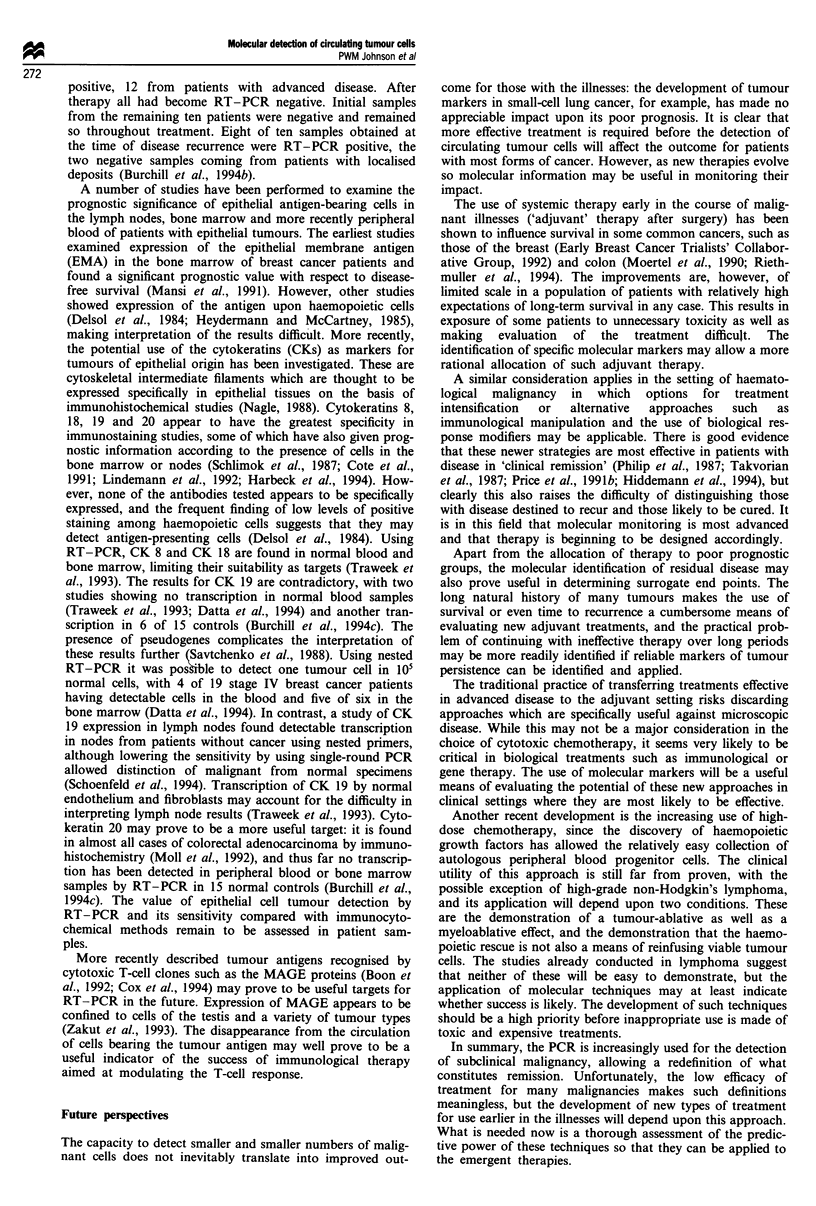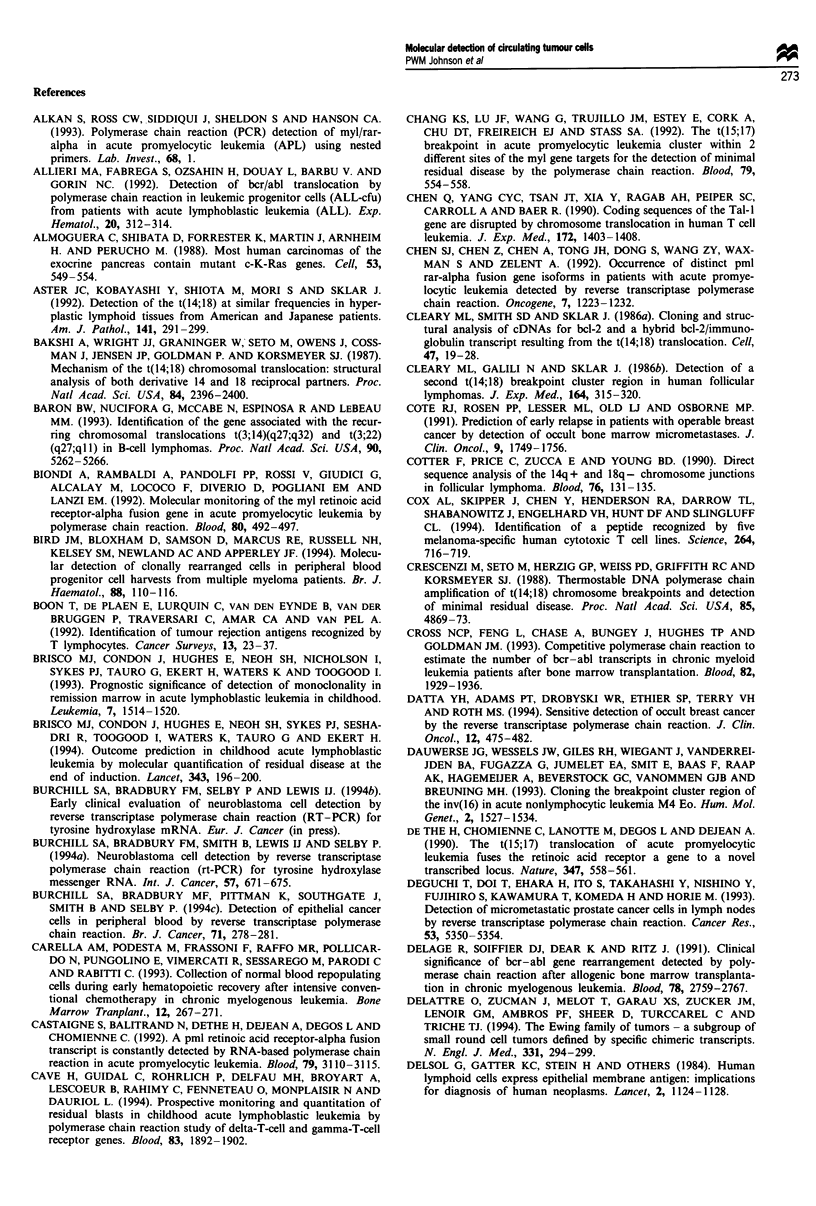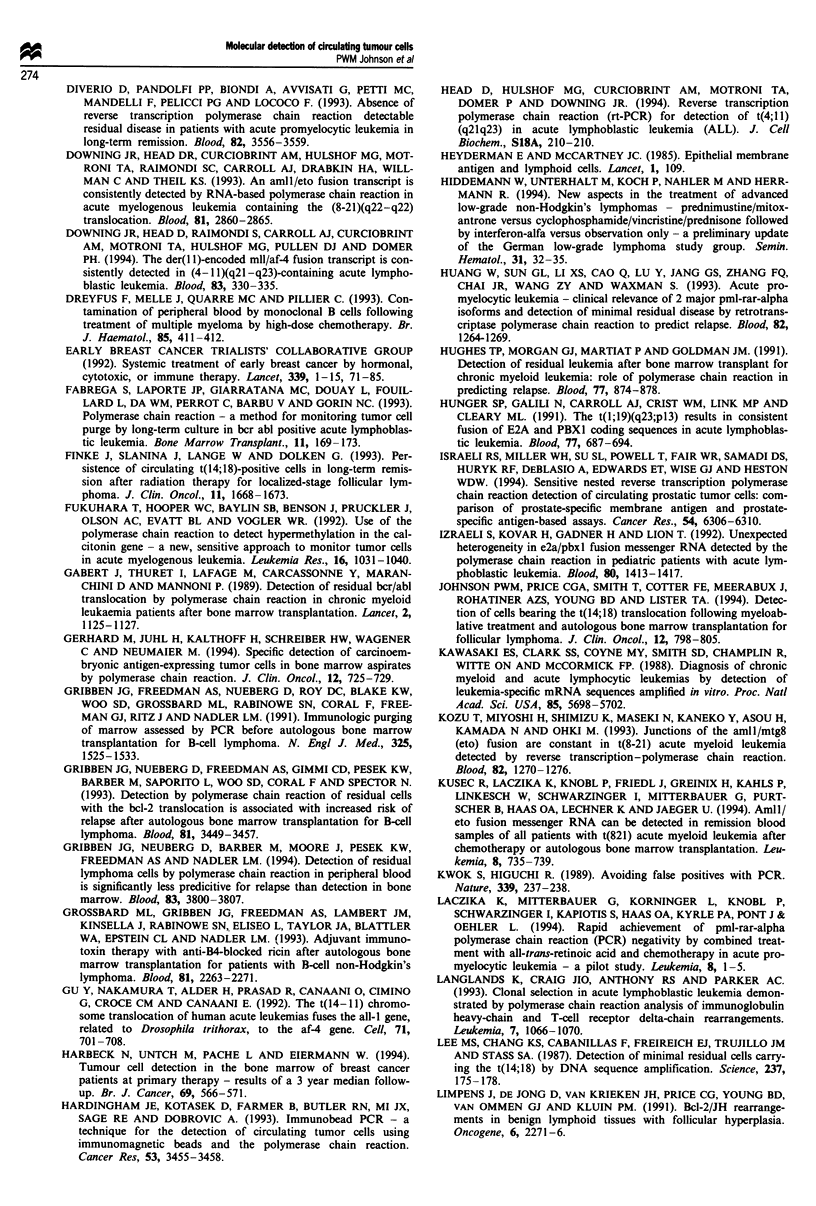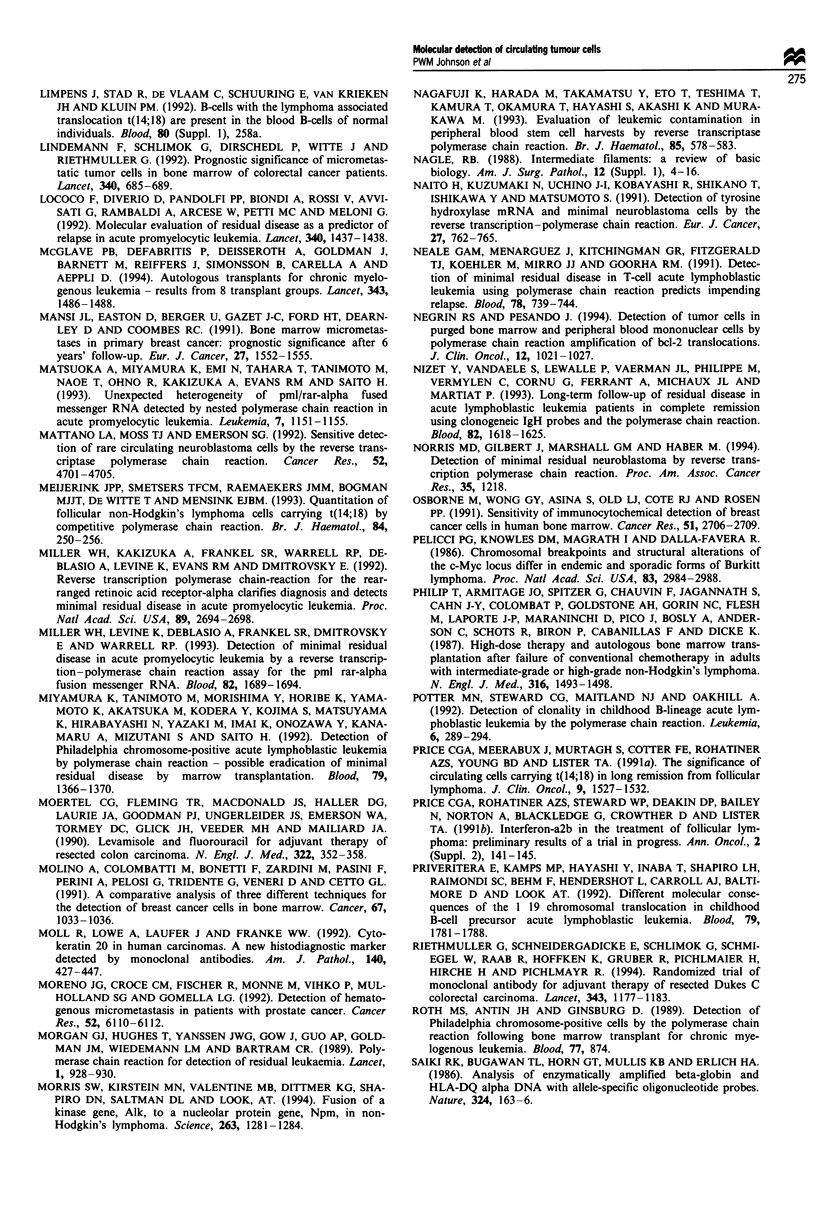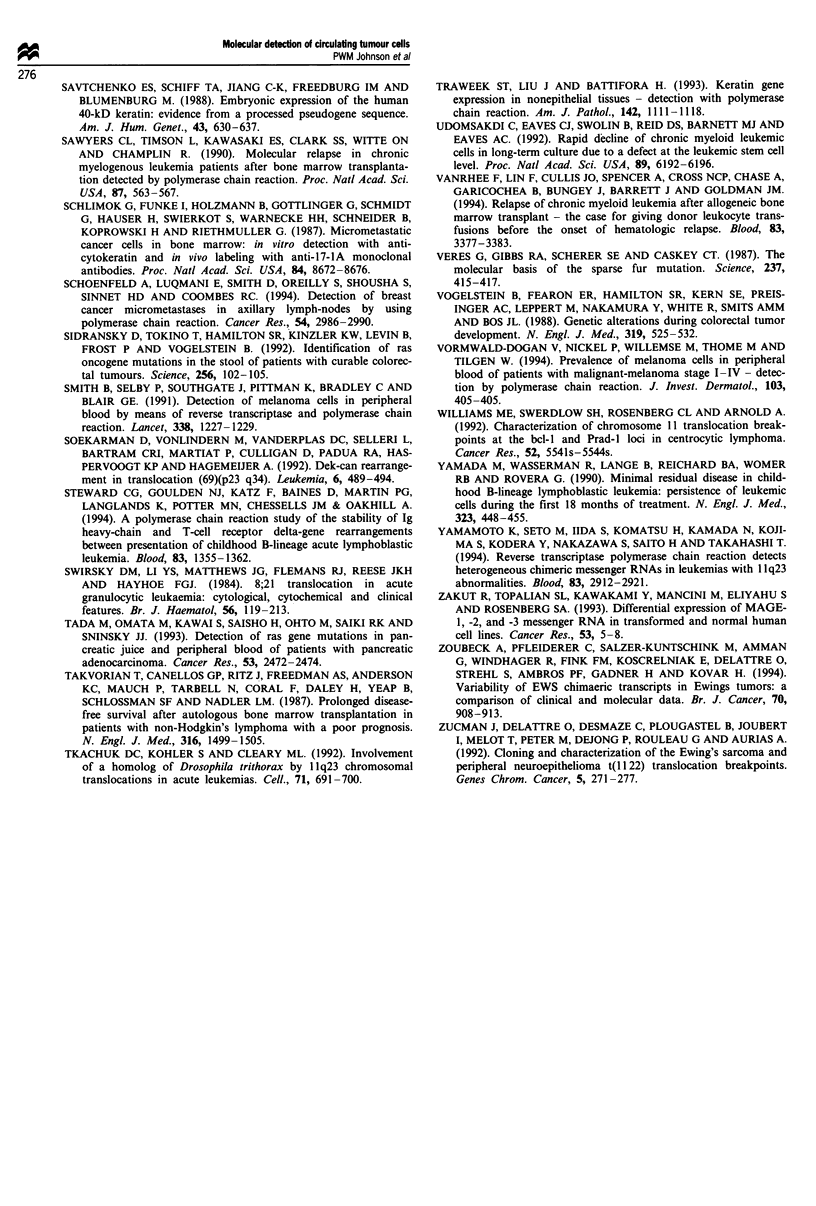# The molecular detection of circulating tumour cells.

**DOI:** 10.1038/bjc.1995.324

**Published:** 1995-08

**Authors:** P. W. Johnson, S. A. Burchill, P. J. Selby

**Affiliations:** ICRF Cancer Medicine Research Unit, St James's University Hospital, Leeds, UK.


British Journal of Cancer (1995) 72, 268-276
© 1995 Stockton Press All rights reserved 0007-0920/95 $12.00

REVIEW

### The molecular detection of circulating tumour cells

PWM Johnson[1], SA Burchill[2] and PJ Selby[1]

[1]ICRF Cancer Medicine Research Unit and [2]Candlelighters Children's Cancer Research Laboratory, St James's University Hospital, Leeds LS9 7TF, UK.

**Keywords:** reverse transcription; polymerase chain reaction; minimal residual disease; molecular diagnostics

Despite advances in the treatment of cancer, recurrence and metastasis continue to pose major problems in clinical management. The relationship between circulating tumour cells and the development of secondary disease is not fully understood. However, a method to detect small numbers of such cells may provide a tool with which to evaluate their role in the disease process, and by implication the possible benefits of eliminating them. One area of convergence between molecular biology and clinical cancer medicine has been in the new methods for detecting systemic spread of tumour cells.

Morphology, flow cytometry and conventional cytogenetics have been used to detect circulating tumour cells at a level of 1 in 100, and the more sensitive method of immunocytochemistry may detect one tumour cell in $10^5$ normals (Molino *et al.*, 1991; Osborne *et al.*, 1991). This technique is, however, dependent upon the availability of antibodies to tumour-associated cell-surface antigens and may be subject to false positives when antibodies cross-react or tumour antigens are presented on host immune cells (Heydermann and McCartney, 1985).

The advent of the polymerase chain reaction (PCR) and the ability to amplify a specific region of DNA between defined oligonucleotide sequences using repeated cycles of denaturation, annealing and extension has made an enormous impact upon nucleic acid analysis (Saiki *et al.*, 1986). By amplification of tumour-specific sequences, the PCR has been shown in a variety of studies to detect one malignant cell in up to $10^7$ normal cells (Mattano *et al.*, 1992; Alkan *et el.*, 1993; Cross *et al.*, 1993; Fabrega *et al.*, 1993; Datta *et al.*, 1994; Gerhard *et al.*, 1994; Negrin and Pesando, 1994). This increases the sensitivity of detection by an order of magnitude when compared with immunocytochemistry.

Studies of PCR amplification of tumour-specific DNA sequences have been possible mainly in haematological malignancies in which consistent and well-characterised molecular abnormalities are present. For solid tumours such abnormalities are uncommon and other strategies are required. We and others have used the amplification of tissue-specific RNA, after reverse transcription, as a marker of solid tumour cells in the blood, thus avoiding the requirement for a DNA sequence abnormality (Smith *et al.*, 1991; Burchill *et al.*, 1994*a*).

### Methods

The choice of target for amplification is evidently determined by the specific characteristics of the malignant cells. Genomic DNA has considerable advantages since archival embedded material can be studied and the extraction process is more robust, but only somatic abnormalities in the tumour cells may usefully be detected in this way. Specific oncogene mutations in genomic DNA may be used to identify malignant cells, although artefactual results owing to errors in polymerisation may complicate the interpretation. To detect low copy numbers of such mutations, mutant-specific primers are necessary to give adequate sensitivity.

Messenger RNA is an increasingly used target for the detection of tumour cells, after production of complementary DNA by reverse transcription (RT-PCR) (Veres *et al.*, 1987). This allows the detection of translocations and other rearrangements which occur within introns as well as providing some tissue specificity according to the genes transcribed in particular cells. The principal limitation to the use of wild-type gene expression for the detection of tumour cells is obviously that the gene should not normally be expressed in peripheral blood cells (or bone marrow or lymph nodes if these are the tissues studied). To study the expression of genes it is important to amplify selectively cDNA produced from the RNA and not contaminating genomic DNA. Removing all DNA from extracted RNA samples can be a problem. Treatment with RNAse-free DNAse and the inclusion of reverse transcriptase negative controls is essential to confirm the specificity of amplification from RNA. Where possible, primers should be selected to span an intron, resulting in the synthesis of different-sized amplification products from the spliced RNA and any contaminating genomic DNA.

The occurrence of false-positive results is a difficulty which is born of the immense power of the technique. The smallest amount of contamination may yield a spurious result, a more difficult problem when there is no distinction in size between the products from different individuals. Only scrupulous attention to laboratory practice and the physical separation of nucleic acid extraction, PCR amplification and the manipulation of amplicons will prevent this (Kwok and Higuchi, 1989).

Unlike more traditional methods of detection, the PCR is difficult to quantitate in a way which yields information about the numbers of positive cells in the population. The paucity of quantitative information has to some extent limited the prognostic power of the technique. The methods devised for quantitation include the addition of different-sized competitor target molecules (Fukuhara *et al.*, 1992; Cross *et al.*, 1993; Meijerink *et al.*, 1993) or the use of serial dilutions (Brisco *et al.*, 1994). The first method makes the assumption that the kinetics of primer/DNA associations is linear in a variety of target/competitor ratios. Although serial dilution of samples is more laborious, comparison of amplification over a range of RNA or DNA concentrations for a target gene compared with a control gene is more reliable providing analysis is made over the exponential range of amplification. However, semi-quantitation of RT-PCR in this way does not allow clear statements regarding tumour cell numbers since the copy number and transcription rate of individual tumour cells will vary between individuals. As with all methodologies, sampling errors assume increasing importance as target cell numbers decline. The RT-PCR in partic-

Correspondence: PWM Johnson
Received 13 January 1995; Revised 10 March 1995; accepted 13 March 1995

ular may be susceptible to failure when transcription is temporarily down-regulated owing to chemotherapy, despite the continued presence of tumour cells.

Increased sensitivity of detection may be achieved by Southern blotting and hybridisation using an oligonucleotide probe to sequences within the amplified segment. This has the advantage of confirming the specificity of the PCR. Alternatively, direct sequencing can be carried out, which may be particularly useful where individuals have unique breakpoints.

Enrichment of samples for tumour cells is a strategy which may become more widespread in the future. Improved immunomagnetic methods of cell sorting make it possible to select for tumour surface antigens before extracting nucleic acids, reducing the amount of background material (Hardingham *et al.*, 1993).

### Malignancies studied

### *Haematological malignancy*

*Immunoglobulin and T-cell receptor gene rearrangements* Valuable information has been obtained in lymphoid malignancies by amplification of clonally rearranged immunoglobulin and T-cell receptor genes. The primers used are complementary to the framework segments of the immunoglobulin variable regions and the consensus joining region for B-cell clones, and the variable and joining regions of the γ- and δ-receptors for T cells. Inevitably, several sets of primers must be tested for each patient in order to determine the most suitable targets. Although the presence of competing polyclonal populations restricts the sensitivity of this method for residual disease, sequencing of the clonal rearrangement and the subsequent use of patient-specific primers can improve this (Potter *et al.*, 1992; Nizet *et al.*, 1993). The technique is finding increasing application in the analysis of haemopoietic progenitor cell harvests used to restore the bone marrow after myeloablative therapy. Initial results in multiple myeloma suggest that peripheral blood progenitor cells often contain populations with clonal IgH rearrangements, although it is not clear whether these contribute to recurrence rates (Dreyfuss *et al.*, 1993; Bird *et al.*, 1994). The rational development of *in vitro* treatments for these harvests by methods such as $CD34^+$ cell selection or immunomagnetic 'purging' will depend upon these analyses for proof of efficacy.

The study of PCR for immunoglobulin and T-cell receptor gene rearrangements has been successfully applied to lymphoblastic leukaemia (ALL) (Yamada *et al.*, 1990). Several groups have demonstrated that the approach is feasible, with up to 90% of childhood ALL patients having amplifiable clonal markers (Steward *et al.*, 1994). In one study of 152 patients, those with a monoclonal band still detectable following induction therapy showed a 57% recurrence rate as compared with 25% for those in whom only polyclonal products were seen. (Brisco *et al.*, 1993). Other smaller studies have confirmed the relationship between recurrence rate and PCR positivity (Neale *et al.*, 1991; Nizet *et al.*, 1993), a relationship which appears to hold for quantitative estimations of the number of residual leukaemic blasts (Brisco *et al.*, 1994). The rate of decline of clonal cell numbers during treatment has also been shown to correlate with the probability of recurrence in some small studies (Nizet *et al.*, 1993; Cave *et al.*, 1994). Unfortunately, there is also a low but definite recurrence rate even for those in whom no clonal population can be identified, possibly owing to clonal evolution (Langlands *et al.*, 1993; Steward *et al.*, 1994).

*Bcl-2/immunoglobulin gene translocations* One of the best-characterised chromosomal rearrangements associated with lymphoma is the t(14;18)(q32;q21), seen particularly in follicular types, which juxtaposes the apoptosis-suppressing *bcl-2* gene with the immunoglobulin heavy-chain genes (Cleary *et al.*, 1986*a*). This translocation is readily detected in genomic DNA using primers complementary to the immunoglobulin joining region consensus and sequences within the major breakpoint region and minor cluster region respectively (Cleary *et al.*, 1986*b*; Lee *et al.*, 1987; Crescenzi *et al.*, 1988). The variety of breakpoints within small clusters, together with the variable insertion of 'N' regions or even fragments of diversity region chromatin (Cotter *et al.*, 1990), results in a considerable size range of amplified PCR products, so that the individual t(14;18) clones may be identified by separation on agarose gels. Sequence analysis has shown that the breakpoint is rarely, if ever, the same in two clones (Bakshi *et al.*, 1987; Cotter *et al.*, 1990; Johnson *et al.*, 1994).

The significance of the detection of cells carrying the t(14;18) is uncertain. Some studies have demonstrated translocations in non-malignant lymphoid tissue (Limpens *et al.*, 1991; Aster *et al.*, 1992) and even normal blood donors (Limpens *et al.*, 1992), while the lymphoma-associated clone may be detected in the blood of patients in remission for several years after both conventional (Price *et al.*, 1991*a*; Finke *et al.*, 1993) and myeloablative therapy (Johnson *et al.*, 1994). There are certainly some data to suggest that failure to remove t(14;18)-bearing cells from autologous bone marrow harvests is associated with earlier recurrence following their use for haemopoietic rescue (Gribben *et al.*, 1991), although this has not been confirmed in other studies (Johnson *et al.*, 1994). Despite the uncertainty regarding the presence of translocation-bearing cells in prolonged remission, the intuitive suggestion that patients are more likely to remain disease free if the clone is eliminated seems to be supported by some data. Patients with PCR-positive bone marrow during follow-up after myeloablative treatment have earlier recurrences (Gribben *et al.*, 1993), although the relationship is less clear in peripheral blood (Gribben *et al.*, 1994). New immunotherapeutic strategies are now being implemented to treat such patients on the basis of PCR results (Grossbard *et al.*, 1993).

A variety of other chromosomal rearrangements which have been described in lymphoma are detectable by PCR. All require mRNA and a reverse transcription step. The translocations described and the genes involved are shown in Table I. The t(8;14), t(2;5) and t(3;14) are all amenable to this approach, although the t(11;14)(q13;q32) of centrocytic lymphoma shows a scattering of breakpoints on chromosome 11 which makes the use of one set of primers inadequate. No studies have yet been carried out using these rearrangements as markers of disease although they have found some use in diagnosis.

*The Philadelphia chromosome* One of the earliest transfers from classical cytogenetics to molecular biology was the identification of the *BCR* and *ABL* genes on either side of the t(9;22)(q34;q11) in chronic myeloid leukaemia (CML) and some cases of ALL. This translocation is now detectable by RT–PCR using different sets of primers for the p190 and p210 variants (Kawasaki *et al.*, 1988). The PCR has been used for monitoring patients with CML following treatment, in particular myeloablative therapy and allogeneic bone marrow transplantation (Gabert *et al.*, 1989; Morgan *et al.*, 1989; Roth *et al.*, 1989; Sawyers *et al.*, 1990). Detection of the translocation over a year from the date of transplantation has been shown to have adverse prognostic significance, while patients who are initially PCR positive before 1 year may often become PCR negative subsequently. In these cases the prognosis is as good as for the consistently PCR-negative group (Delage *et al.*, 1991; Hughes *et al.*, 1991; Cross *et al.*, 1993). The results in Philadelphia-positive ALL are less conclusive owing to the smaller numbers of patients studied. One group has found some patients with no RT–PCR detectable *BCR–ABL* sequences after myeloablative treatment, and the few patients with durable remissions remain PCR negative (Miyamura *et al.*, 1992). Detection of *BCR–ABL* transcripts precedes clinical recurrence, and further treatments such as interferon α or donor leucocyte infusions given at this time may prolong remission (Vanrhee *et al.*, 1994).

**Table I** Chromosomal rearrangements in lymphoma amenable to PCR detection

| *Rearrangement* | *Genes involved* | *Lymphoma type* | *Reference* |
|---|---|---|---|
| t(14;18)(q21;q32) | *Bcl*-2/Ig heavy chain | Follicular | Cleary *et al.* (1986*a*)<br>Crescenzi *et al.* (1988) |
| t(8;14)(q24;q32)<br>t(2;8)<br>t(8;22) | c-*Myc*/Ig genes | Burkitt's | Pelicci *et al.* (1986) |
| t(2;5)(23;q35) | *NPM/Alk* | Large cell anaplastic | Morris *et al.* (1994) |
| t(3;14)(q27;q32) | *Bcl*-6/Ig heavy chain | Diffuse large cell | Baron *et al.* (1993) |
| t(11;14)(q13;q32) | *Bcl*-1/Ig heavy chain | Centrocytic | Williams *et al.* (1992) |

**Table II** Chromosomal rearrangements in leukaemia amenable to PCR detection

| *Rearrangement* | *Genes involved* | *Leukaemia type* | *References* |
|---|---|---|---|
| t(9;22)(q34;q11) | *BCR/Abl* | CML, ALL | Kawasaki *et al.* (1988) |
| t(15;17)(q22;q21) | *RAR-α/PML* | AML(M3) | Biondi *et al.* (1992)<br>Castaigne *et al.* (1992) |
| t(8;21)(q22;q22) | *AML-1/ETO* | AML(M2) | Downing *et al.* (1993)<br>Kozu *et al.* (1993) |
| t(6;9)(p23;q34) | *DEK/CAN* | AML | Soekarman *et al.* (1992) |
| t(1;19)(q23;p13) | *Pbxl/E2A* | Pre-B-ALL | Hunger *et al.* (1991)<br>Izraeli *et al.* (1992)<br>Priveritera *et al.* (1992) |
| t(4;11)(q21;q23)<br>t(9;11)(p22;q23)<br>t(11;19)(q23;p13) | *MLL* on 11q23 | ALL/AML<br>Often paediatric<br>Sometimes secondary | Gu *et al.* (1992)<br>Tkachuk *et al.* (1992)<br>Downing *et al.* (1994)<br>Head *et al.* (1994)<br>Yamamoto *et al.* (1994) |
| Inv(16)(p13;q22) | *CBFβ/MYH11* | AML (M4) | Dauwerse *et al.* (1993) |
| t(1;14)(p34;q11) | *TAL*-1 | T-ALL | Chen *et al.* (1990) |

The use of autologous haemopoietic rescue following high-dose treatment is also being explored in CML and Philadelphia-positive ALL (McGlave *et al.*, 1994). It may be possible to collect t(9;22)-negative peripheral blood progenitor cells if leucapheresis is performed early during granulocyte colony-stimulating factor (G-CSF)-stimulated recovery from cyclophosphamide priming (Carella *et al.*, 1993). Similarly, *in vitro* culture of bone marrow may result in selection of t(9;22)-negative stem cells (Udomsakdi *et al.*, 1992; Fabrega *et al.*, 1993). Tumour cell contamination is a critical factor in both these approaches, and RT–PCR has been used for rapid determination of the quality of reinfused progenitors (Allieri *et al.*, 1992; Nagafuji *et al.*, 1993).

*Retinoic acid receptor gene translocations* The characteristic translocation of acute promyelocytic leukaemia (APML) is the t(15;17)(q22;q11), which transposes the retinoic acid receptor α and *PML* genes (de The *et al.*, 1990). These have been cloned and RT–PCR used to define at least three isoforms, which have been used to monitor residual disease at the end of therapy (Biondi *et al.*, 1992; Castaigne *et al.*, 1992; Miller *et al.*, 1992). The presence of different isoforms complicates the PCR, requiring several sets of primers in order to cover the different breakpoints involved (Chang *et al.*, 1992; Chen *et al.*, 1992; Matsuoka *et al.*, 1993). There has been some suggestion that patients with translocations in exon 3 of *PML* have a worse prognosis than those with intron 6 breakpoints, but this is based upon a small number of observations in patients treated with an unusual type of chemotherapy (Huang *et al.*, 1993).

Detection of the t(15;17) translocation following treatment is a strong indicator of poor prognosis, with recurrences occurring in almost all cases. Those patients in whom the translocation is not detectable after chemotherapy have a high chance of cure (Lococo *et al.*, 1992) and the translocation has not been detected in patients in long-term remission (Diverio *et al.*, 1993). The use of all-*trans* retinoic acid (ATRA) alone does not eliminate the t(15;17) clone even when the clinical response is rapid and apparently complete, and all patients develop recurrent disease if no consolidation chemotherapy is given (Miller *et al.*, 1993). Conversely, the initial use of ATRA in combination with chemotherapy resulted in rapid disappearance of the t(15;17) in a small pilot study (Laczika *et al.*, 1994), giving hope that molecularly guided therapy may be possible in the future.

AML1/ETO *gene translocations* A more recent finding is that the breakpoints in acute myeloid leukaemias characterised by the t(8;21) lie within a single intron of the *AML*1 gene on chromosome 21 and at identical positions in the *ETO* gene on chromosome 8 (Downing *et al.*, 1993; Kozu *et al.*, 1993). Ninety per cent of these cases are of M2 subtype and are generally thought to carry a better than average prognosis (Swirsky *et al.*, 1984). The constant position of the translocation makes it a good target for detection by RT–PCR, and studies are in progress to examine its use as a marker. One group has found persistence of the translocation despite durable complete remissions in seven patients, two of whom had undergone myeloablative therapy and autologous bone marrow transplantation (Kusec *et al.*, 1994). Further studies are awaited to determine whether the translocation really does persist in patients with durable remissions.

As in lymphoma, several consistent chromosomal rearrangements have been characterised at the molecular level in acute leukaemias of various types, all of which may in future be used for the detection of residual disease by RT–PCR. These are shown in Table II.

*Solid tumours*

The cytogenetics of solid tumours are considerably more complex and less well defined than those of haematological malignancy, hence there have been few opportunities to apply PCR techniques to aid diagnosis or monitor disease following treatment (Table III).

The best-characterised abnormalities in solid tumours involve mutations of either oncogenes or tumour-suppressor genes. A common problem in using such mutations as targets for the PCR is the number of different mutation sites and lack of consistency within tumour types. Thus mutations in the p53 gene are found throughout the open reading frame, and although 'hotspots' have been identified even these extend over four exons. However, K-*ras* shows a relatively restricted pattern of mutation in some diseases such as carcinoma of the pancreas (80% of cases) (Almoguera *et al.*, 1988) or colon (50%) (Vogelstein *et al.*, 1988), and recent reports suggest that the use of primers specific to codon 12 mutations may allow detection of tumour cells in pancreatic juice or blood (Hardingham *et al.*, 1993; Tada *et al.*, 1993). PCR followed by phage cloning and hybridisation with radioactive probes has been used to detect *ras* mutations in colorectal cancer cells in faeces (Sidransky *et al.*, 1992) and may be applicable to blood or bone marrow, particularly where samples of the primary tumour are available to confirm the mutation.

*Ewing's sarcoma* One rearrangement which has been characterised is the t(11;22)(q24;q12), found in 85% of Ewing's sarcomas, juxtaposing the *FLI*-1 and *EWS* genes (Zucman *et al.*, 1992). Although the translocation may be detected in genomic DNA, RT–PCR has been the method of choice owing to its increased reliability and the suggestion that different transcripts may be more clinically informative than detection of tumour cells alone (Delattre *et al.*, 1994; Zoubeck *et al.*, 1994). The European Ewing's Sarcoma Study Group is currently evaluating the value of this method in practice.

In the absence of common consistent chromosomal abnormalities in solid tumours, other targets have been sought. The most promising results have been seen with RT–PCR detection of tissue-specific antigens or enzymes.

*RT–PCR of tissue-specific genes* The first tissue-specific enzyme used as a molecular marker was tyrosinase, expressed in pigmented cells as part of the melanin synthesis pathway. Using nested RT–PCR it proved possible to detect one melanoma cell in at least $10^5$ normal cells, although the sensitivity varied according to the levels of tyrosinase transcription in the cell lines used for the experiments. An initial study in seven patients with melanoma showed transcription in the peripheral blood of four, while none was detected in normal controls (Smith *et al.*, 1991). Follow-up studies have been less encouraging, with only three positives among 22 patients with metastatic melanoma (K Pittman *et al.*, in preparation) although recent data from nearly 300 patients with melanoma showed a close correlation between clinical disease stage and the frequency with which tyrosinase transcription could be detected in the blood (Vormwald-Dogan *et al.*, 1994).

Prostate-specific antigen (PSA) mRNA was identified in the peripheral blood (Moreno *et al.*, 1992) and lymph nodes (Deguchi *et al.*, 1993) of small numbers of patients with prostate cancer but not in controls, with a level of sensitivity that appeared better than immunocytochemistry. Further refinement and increased sensitivity have been reported using the recently cloned prostate-specific membrane (PSM) antigen as a target (Israeli *et al.*, 1994). The rate of detection using nested RT–PCR for PSM appeared to be markedly superior following radical prostatectomy, with 68% of patients with negative PSA serology having positive PSM results by PCR as compared with 3% for PSA. In view of difficulties of interpretation for slightly raised levels of PSA and the long natural history of asymptomatic prostate cancer, it is difficult to know whether an increase in sensitivity is likely to contribute usefully to management. The use of such a marker following prostatectomy might be predictive of recurrence, and it will be interesting to see whether trials of hormonal therapy will confer benefit in this setting.

Carcinoembryonic antigen (CEA) expression has been investigated as a marker of gastrointestinal and breast cancer. RT–PCR for CEA was used to detect tumour cells in the bone marrow of 14 of 21 patients, with dilution experiments suggesting a level of sensitivity of 2–5 tumour cells in $10^7$ normal cells (Gerhard *et al.*, 1994). In 56 normal control marrow samples no CEA expression was found, indicating that other members of the CEA gene family expressed on myeloid cells did not interfere. The sensitivity of tumour cell detection by RT–PCR was greater than by immunocytology for CEA or cytokeratins.

In neuroblastoma two different targets for RT–PCR have been analysed. Expression of PGP-9.5, a protein related to neurone-specific enolase (NSE), has been reported as a useful marker (Mattano *et al.*, 1992). The original report described low levels of expression in normal peripheral blood and bone marrow which did not appear to interfere with tumour cell detection. However, a more recent study found levels of PGP-9.5 expression in normal haemopoietic tissue sufficient to exclude its usefulness as a marker (Norris *et al.*, 1994). Expression of tyrosine hydroxylase, the first enzyme in the catecholamine synthesis pathway, has been used to detect neuroblastoma cells in three separate studies. These have shown tyrosine hydroxylase to be the target of choice for examinations of bone marrow (Naito *et al.*, 1991), peripheral blood and peripheral blood progenitor cells (Burchill *et al.*, 1994*a*); Norris *et al.*, 1994). Dilution experiments have demonstrated detection of one tumour cell in $10^5$ normal marrow cells (Naito *et al.*, 1991) or 1 in $10^7$ normal blood cells (Burchill *et al.*, 1994*a*). No transcription of tyrosine hydroxylase has been found in normal haemopoietic tissue. Studies of blood samples from 23 patients showed some correlation with clinical outcome: 13 of 23 presentation samples were

**Table III** Potential targets for detection of solid tumour cells in the circulation by PCR[a] or RT-PCR[b]

| *Tumour type* | *Target gene/antigen* | *References* |
|---|---|---|
| Pancreas/colon | k-*ras* mutation[a] | Sidransky *et al.* (1992)<br>Hardingham *et al.* (1993)<br>Tada *et al.* (1993) |
| Ewing's sarcoma | t(11;22)(q24;q12)[b] | Zucman *et al.* (1992)<br>Delattre *et al.* (1994) |
| Prostate | Prostate-specific antigen[b] | Moreno *et al.* (1992)<br>Deguchi *et al.* (1993) |
| Breast/colorectal | Carcinoembryonic antigen[b] | Gerhard *et al.* (1994) |
| Neuroblastoma | PGP-9.5[b] | Mattano *et al.* (1992) |
| Neuroblastoma | Tyrosine hydroxylase[b] | Naito *et al.* (1991)<br>Burchill *et al.* (1994) |
| Melanoma | Tyrosinase[b] | Smith *et al.* (1991) |
| Epithelial | Cytokeratins[b] | Traweek *et al.* (1993)<br>Burchill *et al.* (1994)<br>Datta *et al.* (1994) |

positive, 12 from patients with advanced disease. After therapy all had become RT–PCR negative. Initial samples from the remaining ten patients were negative and remained so throughout treatment. Eight of ten samples obtained at the time of disease recurrence were RT–PCR positive, the two negative samples coming from patients with localised deposits (Burchill *et al.*, 1994*b*).

A number of studies have been performed to examine the prognostic significance of epithelial antigen-bearing cells in the lymph nodes, bone marrow and more recently peripheral blood of patients with epithelial tumours. The earliest studies examined expression of the epithelial membrane antigen (EMA) in the bone marrow of breast cancer patients and found a significant prognostic value with respect to disease-free survival (Mansi *et al.*, 1991). However, other studies showed expression of the antigen upon haemopoietic cells (Delsol *et al.*, 1984; Heydermann and McCartney, 1985), making interpretation of the results difficult. More recently, the potential use of the cytokeratins (CKs) as markers for tumours of epithelial origin has been investigated. These are cytoskeletal intermediate filaments which are thought to be expressed specifically in epithelial tissues on the basis of immunohistochemical studies (Nagle, 1988). Cytokeratins 8, 18, 19 and 20 appear to have the greatest specificity in immunostaining studies, some of which have also given prognostic information according to the presence of cells in the bone marrow or nodes (Schlimok *et al.*, 1987; Cote *et al.*, 1991; Lindemann *et al.*, 1992; Harbeck *et al.*, 1994). However, none of the antibodies tested appears to be specifically expressed, and the frequent finding of low levels of positive staining among haemopoietic cells suggests that they may detect antigen-presenting cells (Delsol *et al.*, 1984). Using RT–PCR, CK 8 and CK 18 are found in normal blood and bone marrow, limiting their suitability as targets (Traweek *et al.*, 1993). The results for CK 19 are contradictory, with two studies showing no transcription in normal blood samples (Traweek *et al.*, 1993; Datta *et al.*, 1994) and another transcription in 6 of 15 controls (Burchill *et al.*, 1994*c*). The presence of pseudogenes complicates the interpretation of these results further (Savtchenko *et al.*, 1988). Using nested RT–PCR it was possible to detect one tumour cell in $10^5$ normal cells, with 4 of 19 stage IV breast cancer patients having detectable cells in the blood and five of six in the bone marrow (Datta *et al.*, 1994). In contrast, a study of CK 19 expression in lymph nodes found detectable transcription in nodes from patients without cancer using nested primers, although lowering the sensitivity by using single-round PCR allowed distinction of malignant from normal specimens (Schoenfeld *et al.*, 1994). Transcription of CK 19 by normal endothelium and fibroblasts may account for the difficulty in interpreting lymph node results (Traweek *et al.*, 1993). Cytokeratin 20 may prove to be a more useful target: it is found in almost all cases of colorectal adenocarcinoma by immunohistochemistry (Moll *et al.*, 1992), and thus far no transcription has been detected in peripheral blood or bone marrow samples by RT–PCR in 15 normal controls (Burchill *et al.*, 1994*c*). The value of epithelial cell tumour detection by RT–PCR and its sensitivity compared with immunocytochemical methods remain to be assessed in patient samples.

More recently described tumour antigens recognised by cytotoxic T-cell clones such as the MAGE proteins (Boon *et al.*, 1992; Cox *et al.*, 1994) may prove to be useful targets for RT–PCR in the future. Expression of MAGE appears to be confined to cells of the testis and a variety of tumour types (Zakut *et al.*, 1993). The disappearance from the circulation of cells bearing the tumour antigen may well prove to be a useful indicator of the success of immunological therapy aimed at modulating the T-cell response.

### Future perspectives

The capacity to detect smaller and smaller numbers of malignant cells does not inevitably translate into improved outcome for those with the illnesses: the development of tumour markers in small-cell lung cancer, for example, has made no appreciable impact upon its poor prognosis. It is clear that more effective treatment is required before the detection of circulating tumour cells will affect the outcome for patients with most forms of cancer. However, as new therapies evolve so molecular information may be useful in monitoring their impact.

The use of systemic therapy early in the course of malignant illnesses ('adjuvant' therapy after surgery) has been shown to influence survival in some common cancers, such as those of the breast (Early Breast Cancer Trialists' Collaborative Group, 1992) and colon (Moertel *et al.*, 1990; Riethmuller *et al.*, 1994). The improvements are, however, of limited scale in a population of patients with relatively high expectations of long-term survival in any case. This results in exposure of some patients to unnecessary toxicity as well as making evaluation of the treatment difficult. The identification of specific molecular markers may allow a more rational allocation of such adjuvant therapy.

A similar consideration applies in the setting of haematological malignancy in which options for treatment intensification or alternative approaches such as immunological manipulation and the use of biological response modifiers may be applicable. There is good evidence that these newer strategies are most effective in patients with disease in 'clinical remission' (Philip *et al.*, 1987; Takvorian *et al.*, 1987; Price *et al.*, 1991*b*; Hiddemann *et al.*, 1994), but clearly this also raises the difficulty of distinguishing those with disease destined to recur and those likely to be cured. It is in this field that molecular monitoring is most advanced and that therapy is beginning to be designed accordingly.

Apart from the allocation of therapy to poor prognostic groups, the molecular identification of residual disease may also prove useful in determining surrogate end points. The long natural history of many tumours makes the use of survival or even time to recurrence a cumbersome means of evaluating new adjuvant treatments, and the practical problem of continuing with ineffective therapy over long periods may be more readily identified if reliable markers of tumour persistence can be identified and applied.

The traditional practice of transferring treatments effective in advanced disease to the adjuvant setting risks discarding approaches which are specifically useful against microscopic disease. While this may not be a major consideration in the choice of cytotoxic chemotherapy, it seems very likely to be critical in biological treatments such as immunological or gene therapy. The use of molecular markers will be a useful means of evaluating the potential of these new approaches in clinical settings where they are most likely to be effective.

Another recent development is the increasing use of high-dose chemotherapy, since the discovery of haemopoietic growth factors has allowed the relatively easy collection of autologous peripheral blood progenitor cells. The clinical utility of this approach is still far from proven, with the possible exception of high-grade non-Hodgkin's lymphoma, and its application will depend upon two conditions. These are the demonstration of a tumour-ablative as well as a myeloablative effect, and the demonstration that the haemopoietic rescue is not also a means of reinfusing viable tumour cells. The studies already conducted in lymphoma suggest that neither of these will be easy to demonstrate, but the application of molecular techniques may at least indicate whether success is likely. The development of such techniques should be a high priority before inappropriate use is made of toxic and expensive treatments.

In summary, the PCR is increasingly used for the detection of subclinical malignancy, allowing a redefinition of what constitutes remission. Unfortunately, the low efficacy of treatment for many malignancies makes such definitions meaningless, but the development of new types of treatment for use earlier in the illnesses will depend upon this approach. What is needed now is a thorough assessment of the predictive power of these techniques so that they can be applied to the emergent therapies.

### References

ALKAN S, ROSS CW, SIDDIQUI J, SHELDON S AND HANSON CA. (1993). Polymerase chain reaction (PCR) detection of myl/rar-alpha in acute promyelocytic leukemia (APL) using nested primers. *Lab. Invest.*, **68**, 1.

ALLIERI MA, FABREGA S, OZSAHIN H, DOUAY L, BARBU V. AND GORIN NC. (1992). Detection of bcr/abl translocation by polymerase chain reaction in leukemic progenitor cells (ALL-cfu) from patients with acute lymphoblastic leukemia (ALL). *Exp. Hematol.*, **20**, 312–314.

ALMOGUERA C, SHIBATA D, FORRESTER K, MARTIN J, ARNHEIM H. AND PERUCHO M. (1988). Most human carcinomas of the exocrine pancreas contain mutant c-K-Ras genes. *Cell*, **53**, 549–554.

ASTER JC, KOBAYASHI Y, SHIOTA M, MORI S AND SKLAR J. (1992). Detection of the t(14;18) at similar frequencies in hyperplastic lymphoid tissues from American and Japanese patients. *Am. J. Pathol.*, **141**, 291–299.

BAKSHI A, WRIGHT JJ, GRANINGER W, SETO M, OWENS J, COSSMAN J, JENSEN JP, GOLDMAN P. AND KORSMEYER SJ. (1987). Mechanism of the t(14;18) chromosomal translocation: structural analysis of both derivative 14 and 18 reciprocal partners. *Proc. Natl Acad. Sci. USA*, **84**, 2396–2400.

BARON BW, NUCIFORA G, MCCABE N, ESPINOSA R AND LEBEAU MM. (1993). Identification of the gene associated with the recurring chromosomal translocations t(3;14)(q27;q32) and t(3;22)(q27;q11) in B-cell lymphomas. *Proc. Natl Acad. Sci. USA*, **90**, 5262–5266.

BIONDI A, RAMBALDI A, PANDOLFI PP, ROSSI V, GIUDICI G, ALCALAY M, LOCOCO F, DIVERIO D, POGLIANI EM AND LANZI EM. (1992). Molecular monitoring of the myl retinoic acid receptor-alpha fusion gene in acute promyelocytic leukemia by polymerase chain reaction. *Blood*, **80**, 492–497.

BIRD JM, BLOXHAM D, SAMSON D, MARCUS RE, RUSSELL NH, KELSEY SM, NEWLAND AC AND APPERLEY JF. (1994). Molecular detection of clonally rearranged cells in peripheral blood progenitor cell harvests from multiple myeloma patients. *Br. J. Haematol.*, **88**, 110–116.

BOON T, DE PLAEN E, LURQUIN C, VAN DEN EYNDE B, VAN DER BRUGGEN P, TRAVERSARI C, AMAR CA AND VAN PEL A. (1992). Identification of tumour rejection antigens recognized by T lymphocytes. *Cancer Surveys*, **13**, 23–37.

BRISCO MJ, CONDON J, HUGHES E, NEOH SH, NICHOLSON I, SYKES PJ, TAURO G, EKERT H, WATERS K AND TOOGOOD I. (1993). Prognostic significance of detection of monoclonality in remission marrow in acute lymphoblastic leukemia in childhood. *Leukemia*, **7**, 1514–1520.

BRISCO MJ, CONDON J, HUGHES E, NEOH SH, SYKES PJ, SESHADRI R, TOOGOOD I, WATERS K, TAURO G AND EKERT H. (1994). Outcome prediction in childhood acute lymphoblastic leukemia by molecular quantification of residual disease at the end of induction. *Lancet*, **343**, 196–200.

BURCHILL SA, BRADBURY FM, SELBY P AND LEWIS IJ. (1994*b*). Early clinical evaluation of neuroblastoma cell detection by reverse transcriptase polymerase chain reaction (RT-PCR) for tyrosine hydroxylase mRNA. *Eur. J. Cancer* (in press).

BURCHILL SA, BRADBURY FM, SMITH B, LEWIS IJ AND SELBY P. (1994*a*). Neuroblastoma cell detection by reverse transcriptase polymerase chain reaction (rt-PCR) for tyrosine hydroxylase messenger RNA. *Int. J. Cancer*, **57**, 671–675.

BURCHILL SA, BRADBURY MF, PITTMAN K, SOUTHGATE J, SMITH B AND SELBY P. (1994*c*). Detection of epithelial cancer cells in peripheral blood by reverse transcriptase polymerase chain reaction. *Br. J. Cancer*, **71**, 278–281.

CARELLA AM, PODESTA M, FRASSONI F, RAFFO MR, POLLICARDO N, PUNGOLINO E, VIMERCATI R, SESSAREGO M, PARODI C AND RABITTI C. (1993). Collection of normal blood repopulating cells during early hematopoietic recovery after intensive conventional chemotherapy in chronic myelogenous leukemia. *Bone Marrow Tranplant.*, **12**, 267–271.

CASTAIGNE S, BALITRAND N, DETHE H, DEJEAN A, DEGOS L AND CHOMIENNE C. (1992). A pml retinoic acid receptor-alpha fusion transcript is constantly detected by RNA-based polymerase chain reaction in acute promyelocytic leukemia. *Blood*, **79**, 3110–3115.

CAVE H, GUIDAL C, ROHRLICH P, DELFAU MH, BROYART A, LESCOEUR B, RAHIMY C, FENNETEAU O, MONPLAISIR N AND DAURIOL L. (1994). Prospective monitoring and quantitation of residual blasts in childhood acute lymphoblastic leukemia by polymerase chain reaction study of delta-T-cell and gamma-T-cell receptor genes. *Blood*, **83**, 1892–1902.

CHANG KS, LU JF, WANG G, TRUJILLO JM, ESTEY E, CORK A, CHU DT, FREIREICH EJ AND STASS SA. (1992). The t(15;17) breakpoint in acute promyelocytic leukemia cluster within 2 different sites of the myl gene targets for the detection of minimal residual disease by the polymerase chain reaction. *Blood*, **79**, 554–558.

CHEN Q, YANG CYC, TSAN JT, XIA Y, RAGAB AH, PEIPER SC, CARROLL A AND BAER R. (1990). Coding sequences of the Tal-1 gene are disrupted by chromosome translocation in human T cell leukemia. *J. Exp. Med.*, **172**, 1403–1408.

CHEN SJ, CHEN Z, CHEN A, TONG JH, DONG S, WANG ZY, WAXMAN S AND ZELENT A. (1992). Occurrence of distinct pml rar-alpha fusion gene isoforms in patients with acute promyelocytic leukemia detected by reverse transcriptase polymerase chain reaction. *Oncogene*, **7**, 1223–1232.

CLEARY ML, SMITH SD AND SKLAR J. (1986*a*). Cloning and structural analysis of cDNAs for bcl-2 and a hybrid bcl-2/immunoglobulin transcript resulting from the t(14;18) translocation. *Cell*, **47**, 19–28.

CLEARY ML, GALILI N AND SKLAR J. (1986*b*). Detection of a second t(14;18) breakpoint cluster region in human follicular lymphomas. *J. Exp. Med.*, **164**, 315–320.

COTE RJ, ROSEN PP, LESSER ML, OLD LJ AND OSBORNE MP. (1991). Prediction of early relapse in patients with operable breast cancer by detection of occult bone marrow micrometastases. *J. Clin. Oncol.*, **9**, 1749–1756.

COTTER F, PRICE C, ZUCCA E AND YOUNG BD. (1990). Direct sequence analysis of the 14q+ and 18q− chromosome junctions in follicular lymphoma. *Blood*, **76**, 131–135.

COX AL, SKIPPER J, CHEN Y, HENDERSON RA, DARROW TL, SHABANOWITZ J, ENGELHARD VH, HUNT DF AND SLINGLUFF CL. (1994). Identification of a peptide recognized by five melanoma-specific human cytotoxic T cell lines. *Science*, **264**, 716–719.

CRESCENZI M, SETO M, HERZIG GP, WEISS PD, GRIFFITH RC AND KORSMEYER SJ. (1988). Thermostable DNA polymerase chain amplification of t(14;18) chromosome breakpoints and detection of minimal residual disease. *Proc. Natl Acad. Sci. USA*, **85**, 4869–73.

CROSS NCP, FENG L, CHASE A, BUNGEY J, HUGHES TP AND GOLDMAN JM. (1993). Competitive polymerase chain reaction to estimate the number of bcr–abl transcripts in chronic myeloid leukemia patients after bone marrow transplantation. *Blood*, **82**, 1929–1936.

DATTA YH, ADAMS PT, DROBYSKI WR, ETHIER SP, TERRY VH AND ROTH MS. (1994). Sensitive detection of occult breast cancer by the reverse transcriptase polymerase chain reaction. *J. Clin. Oncol.*, **12**, 475–482.

DAUWERSE JG, WESSELS JW, GILES RH, WIEGANT J, VANDERREIJDEN BA, FUGAZZA G, JUMELET EA, SMIT E, BAAS F, RAAP AK, HAGEMEIJER A, BEVERSTOCK GC, VANOMMEN GJB AND BREUNING MH. (1993). Cloning the breakpoint cluster region of the inv(16) in acute nonlymphocytic leukemia M4 Eo. *Hum. Mol. Genet.*, **2**, 1527–1534.

DE THE H, CHOMIENNE C, LANOTTE M, DEGOS L AND DEJEAN A. (1990). The t(15;17) translocation of acute promyelocytic leukemia fuses the retinoic acid receptor a gene to a novel transcribed locus. *Nature*, **347**, 558–561.

DEGUCHI T, DOI T, EHARA H, ITO S, TAKAHASHI Y, NISHINO Y, FUJIHIRO S, KAWAMURA T, KOMEDA H AND HORIE M. (1993). Detection of micrometastatic prostate cancer cells in lymph nodes by reverse transcriptase polymerase chain reaction. *Cancer Res.*, **53**, 5350–5354.

DELAGE R, SOIFFIER DJ, DEAR K AND RITZ J. (1991). Clinical significance of bcr–abl gene rearrangement detected by polymerase chain reaction after allogenic bone marrow transplantation in chronic myelogenous leukemia. *Blood*, **78**, 2759–2767.

DELATTRE O, ZUCMAN J, MELOT T, GARAU XS, ZUCKER JM, LENOIR GM, AMBROS PF, SHEER D, TURCCAREL C AND TRICHE TJ. (1994). The Ewing family of tumors – a subgroup of small round cell tumors defined by specific chimeric transcripts. *N. Engl. J. Med.*, **331**, 294–299.

DELSOL G, GATTER KC, STEIN H AND OTHERS (1984). Human lymphoid cells express epithelial membrane antigen: implications for diagnosis of human neoplasms. *Lancet*, **2**, 1124–1128.

DIVERIO D, PANDOLFI PP, BIONDI A, AVVISATI G, PETTI MC, MANDELLI F, PELICCI PG AND LOCOCO F. (1993). Absence of reverse transcription polymerase chain reaction detectable residual disease in patients with acute promyelocytic leukemia in long-term remission. *Blood*, **82**, 3556–3559.

DOWNING JR, HEAD DR, CURCIOBRINT AM, HULSHOF MG, MOTRONI TA, RAIMONDI SC, CARROLL AJ, DRABKIN HA, WILLMAN C AND THEIL KS. (1993). An aml1/eto fusion transcript is consistently detected by RNA-based polymerase chain reaction in acute myelogenous leukemia containing the (8-21)(q22–q22) translocation. *Blood*, **81**, 2860–2865.

DOWNING JR, HEAD D, RAIMONDI S, CARROLL AJ, CURCIOBRINT AM, MOTRONI TA, HULSHOF MG, PULLEN DJ AND DOMER PH. (1994). The der(11)-encoded mll/af-4 fusion transcript is consistently detected in (4–11)(q21–q23)-containing acute lymphoblastic leukemia. *Blood*, **83**, 330–335.

DREYFUS F, MELLE J, QUARRE MC AND PILLIER C. (1993). Contamination of peripheral blood by monoclonal B cells following treatment of multiple myeloma by high-dose chemotherapy. *Br. J. Haematol.*, **85**, 411–412.

EARLY BREAST CANCER TRIALISTS' COLLABORATIVE GROUP (1992). Systemic treatment of early breast cancer by hormonal, cytotoxic, or immune therapy. *Lancet*, **339**, 1–15, 71–85.

FABREGA S, LAPORTE JP, GIARRATANA MC, DOUAY L, FOUILLARD L, DA WM, PERROT C, BARBU V AND GORIN NC. (1993). Polymerase chain reaction – a method for monitoring tumor cell purge by long-term culture in bcr abl positive acute lymphoblastic leukemia. *Bone Marrow Transplant.*, **11**, 169–173.

FINKE J, SLANINA J, LANGE W AND DOLKEN G. (1993). Persistence of circulating t(14;18)-positive cells in long-term remission after radiation therapy for localized-stage follicular lymphoma. *J. Clin. Oncol.*, **11**, 1668–1673.

FUKUHARA T, HOOPER WC, BAYLIN SB, BENSON J, PRUCKLER J, OLSON AC, EVATT BL AND VOGLER WR. (1992). Use of the polymerase chain reaction to detect hypermethylation in the calcitonin gene – a new, sensitive approach to monitor tumor cells in acute myelogenous leukemia. *Leukemia Res.*, **16**, 1031–1040.

GABERT J, THURET I, LAFAGE M, CARCASSONNE Y, MARANCHINI D AND MANNONI P. (1989). Detection of residual bcr/abl translocation by polymerase chain reaction in chronic myeloid leukaemia patients after bone marrow transplantation. *Lancet*, **2**, 1125–1127.

GERHARD M, JUHL H, KALTHOFF H, SCHREIBER HW, WAGENER C AND NEUMAIER M. (1994). Specific detection of carcinoembryonic antigen-expressing tumor cells in bone marrow aspirates by polymerase chain reaction. *J. Clin. Oncol.*, **12**, 725–729.

GRIBBEN JG, FREEDMAN AS, NUEBERG D, ROY DC, BLAKE KW, WOO SD, GROSSBARD ML, RABINOWE SN, CORAL F, FREEMAN GJ, RITZ J AND NADLER LM. (1991). Immunologic purging of marrow assessed by PCR before autologous bone marrow transplantation for B-cell lymphoma. *N. Engl J. Med.*, **325**, 1525–1533.

GRIBBEN JG, NUEBERG D, FREEDMAN AS, GIMMI CD, PESEK KW, BARBER M, SAPORITO L, WOO SD, CORAL F AND SPECTOR N. (1993). Detection by polymerase chain reaction of residual cells with the bcl-2 translocation is associated with increased risk of relapse after autologous bone marrow transplantation for B-cell lymphoma. *Blood*, **81**, 3449–3457.

GRIBBEN JG, NEUBERG D, BARBER M, MOORE J, PESEK KW, FREEDMAN AS AND NADLER LM. (1994). Detection of residual lymphoma cells by polymerase chain reaction in peripheral blood is significantly less predicitive for relapse than detection in bone marrow. *Blood*, **83**, 3800–3807.

GROSSBARD ML, GRIBBEN JG, FREEDMAN AS, LAMBERT JM, KINSELLA J, RABINOWE SN, ELISEO L, TAYLOR JA, BLATTLER WA, EPSTEIN CL AND NADLER LM. (1993). Adjuvant immunotoxin therapy with anti-B4-blocked ricin after autologous bone marrow transplantation for patients with B-cell non-Hodgkin's lymphoma. *Blood*, **81**, 2263–2271.

GU Y, NAKAMURA T, ALDER H, PRASAD R, CANAANI O, CIMINO G, CROCE CM AND CANAANI E. (1992). The t(14–11) chromosome translocation of human acute leukemias fuses the all-1 gene, related to *Drosophila trithorax*, to the af-4 gene. *Cell*, **71**, 701–708.

HARBECK N, UNTCH M, PACHE L AND EIERMANN W. (1994). Tumour cell detection in the bone marrow of breast cancer patients at primary therapy – results of a 3 year median follow-up. *Br. J. Cancer*, **69**, 566–571.

HARDINGHAM JE, KOTASEK D, FARMER B, BUTLER RN, MI JX, SAGE RE AND DOBROVIC A. (1993). Immunobead PCR – a technique for the detection of circulating tumor cells using immunomagnetic beads and the polymerase chain reaction. *Cancer Res*, **53**, 3455–3458.

HEAD D, HULSHOF MG, CURCIOBRINT AM, MOTRONI TA, DOMER P AND DOWNING JR. (1994). Reverse transcription polymerase chain reaction (rt-PCR) for detection of t(4;11) (q21q23) in acute lymphoblastic leukemia (ALL). *J. Cell Biochem.*, **S18A**, 210–210.

HEYDERMAN E AND MCCARTNEY JC. (1985). Epithelial membrane antigen and lymphoid cells. *Lancet*, **1**, 109.

HIDDEMANN W, UNTERHALT M, KOCH P, NAHLER M AND HERRMANN R. (1994). New aspects in the treatment of advanced low-grade non-Hodgkin's lymphomas – prednimustine/mitoxantrone versus cyclophosphamide/vincristine/prednisone followed by interferon-alfa versus observation only – a preliminary update of the German low-grade lymphoma study group. *Semin. Hematol.*, **31**, 32–35.

HUANG W, SUN GL, LI XS, CAO Q, LU Y, JANG GS, ZHANG FQ, CHAI JR, WANG ZY AND WAXMAN S. (1993). Acute promyelocytic leukemia – clinical relevance of 2 major pml-rar-alpha isoforms and detection of minimal residual disease by retrotranscriptase polymerase chain reaction to predict relapse. *Blood*, **82**, 1264-1269.

HUGHES TP, MORGAN GJ, MARTIAT P AND GOLDMAN JM. (1991). Detection of residual leukemia after bone marrow transplant for chronic myeloid leukemia: role of polymerase chain reaction in predicting relapse. *Blood*, **77**, 874–878.

HUNGER SP, GALILI N, CARROLL AJ, CRIST WM, LINK MP AND CLEARY ML. (1991). The t(1;19)(q23;p13) results in consistent fusion of E2A and PBX1 coding sequences in acute lymphoblastic leukemia. *Blood*, **77**, 687–694.

ISRAELI RS, MILLER WH, SU SL, POWELL T, FAIR WR, SAMADI DS, HURYK RF, DEBLASIO A, EDWARDS ET, WISE GJ AND HESTON WDW. (1994). Sensitive nested reverse transcription polymerase chain reaction detection of circulating prostatic tumor cells: comparison of prostate-specific membrane antigen and prostate-specific antigen-based assays. *Cancer Res.*, **54**, 6306–6310.

IZRAELI S, KOVAR H, GADNER H AND LION T. (1992). Unexpected heterogeneity in e2a/pbx1 fusion messenger RNA detected by the polymerase chain reaction in pediatric patients with acute lymphoblastic leukemia. *Blood*, **80**, 1413–1417.

JOHNSON PWM, PRICE CGA, SMITH T, COTTER FE, MEERABUX J, ROHATINER AZS, YOUNG BD AND LISTER TA. (1994). Detection of cells bearing the t(14;18) translocation following myeloablative treatment and autologous bone marrow transplantation for follicular lymphoma. *J. Clin. Oncol.*, **12**, 798–805.

KAWASAKI ES, CLARK SS, COYNE MY, SMITH SD, CHAMPLIN R, WITTE ON AND MCCORMICK FP. (1988). Diagnosis of chronic myeloid and acute lymphocytic leukemias by detection of leukemia-specific mRNA sequences amplified *in vitro*. *Proc. Natl Acad. Sci. USA*, **85**, 5698–5702.

KOZU T, MIYOSHI H, SHIMIZU K, MASEKI N, KANEKO Y, ASOU H, KAMADA N AND OHKI M. (1993). Junctions of the aml1/mtg8 (eto) fusion are constant in t(8-21) acute myeloid leukemia detected by reverse transcription–polymerase chain reaction. *Blood*, **82**, 1270–1276.

KUSEC R, LACZIKA K, KNOBL P, FRIEDL J, GREINIX H, KAHLS P, LINKESCH W, SCHWARZINGER I, MITTERBAUER G, PURTSCHER B, HAAS OA, LECHNER K AND JAEGER U. (1994). Aml1/eto fusion messenger RNA can be detected in remission blood samples of all patients with t(821) acute myeloid leukemia after chemotherapy or autologous bone marrow transplantation. *Leukemia*, **8**, 735–739.

KWOK S, HIGUCHI R. (1989). Avoiding false positives with PCR. *Nature*, **339**, 237–238.

LACZIKA K, MITTERBAUER G, KORNINGER L, KNOBL P, SCHWARZINGER I, KAPIOTIS S, HAAS OA, KYRLE PA, PONT J & OEHLER L. (1994). Rapid achievement of pml-rar-alpha polymerase chain reaction (PCR) negativity by combined treatment with all-*trans*-retinoic acid and chemotherapy in acute promyelocytic leukemia – a pilot study. *Leukemia*, **8**, 1–5.

LANGLANDS K, CRAIG JIO, ANTHONY RS AND PARKER AC. (1993). Clonal selection in acute lymphoblastic leukemia demonstrated by polymerase chain reaction analysis of immunoglobulin heavy-chain and T-cell receptor delta-chain rearrangements. *Leukemia*, **7**, 1066–1070.

LEE MS, CHANG KS, CABANILLAS F, FREIREICH EJ, TRUJILLO JM AND STASS SA. (1987). Detection of minimal residual cells carrying the t(14;18) by DNA sequence amplification. *Science*, **237**, 175–178.

LIMPENS J, DE JONG D, VAN KRIEKEN JH, PRICE CG, YOUNG BD, VAN OMMEN GJ AND KLUIN PM. (1991). Bcl-2/JH rearrangements in benign lymphoid tissues with follicular hyperplasia. *Oncogene*, **6**, 2271–6.

LIMPENS J, STAD R, DE VLAAM C, SCHUURING E, VAN KRIEKEN JH AND KLUIN PM. (1992). B-cells with the lymphoma associated translocation t(14;18) are present in the blood B-cells of normal individuals. *Blood*, **80** (Suppl. 1), 258a.

LINDEMANN F, SCHLIMOK G, DIRSCHEDL P, WITTE J AND RIETHMULLER G. (1992). Prognostic significance of micrometastatic tumor cells in bone marrow of colorectal cancer patients. *Lancet*, **340**, 685–689.

LOCOCO F, DIVERIO D, PANDOLFI PP, BIONDI A, ROSSI V, AVVISATI G, RAMBALDI A, ARCESE W, PETTI MC AND MELONI G. (1992). Molecular evaluation of residual disease as a predictor of relapse in acute promyelocytic leukemia. *Lancet*, **340**, 1437–1438.

MCGLAVE PB, DEFABRITIS P, DEISSEROTH A, GOLDMAN J, BARNETT M, REIFFERS J, SIMONSSON B, CARELLA A AND AEPPLI D. (1994). Autologous transplants for chronic myelogenous leukemia – results from 8 transplant groups. *Lancet*, **343**, 1486–1488.

MANSI JL, EASTON D, BERGER U, GAZET J-C, FORD HT, DEARNLEY D AND COOMBES RC. (1991). Bone marrow micrometastases in primary breast cancer: prognostic significance after 6 years' follow-up. *Eur. J. Cancer*, **27**, 1552–1555.

MATSUOKA A, MIYAMURA K, EMI N, TAHARA T, TANIMOTO M, NAOE T, OHNO R, KAKIZUKA A, EVANS RM AND SAITO H. (1993). Unexpected heterogeneity of pml/rar-alpha fused messenger RNA detected by nested polymerase chain reaction in acute promyelocytic leukemia. *Leukemia*, **7**, 1151–1155.

MATTANO LA, MOSS TJ AND EMERSON SG. (1992). Sensitive detection of rare circulating neuroblastoma cells by the reverse transcriptase polymerase chain reaction. *Cancer Res.*, **52**, 4701–4705.

MEIJERINK JPP, SMETSERS TFCM, RAEMAEKERS JMM, BOGMAN MJJT, DE WITTE T AND MENSINK EJBM. (1993). Quantitation of follicular non-Hodgkin's lymphoma cells carrying t(14;18) by competitive polymerase chain reaction. *Br. J. Haematol.*, **84**, 250–256.

MILLER WH, KAKIZUKA A, FRANKEL SR, WARRELL RP, DEBLASIO A, LEVINE K, EVANS RM AND DMITROVSKY E. (1992). Reverse transcription polymerase chain-reaction for the rearranged retinoic acid receptor-alpha clarifies diagnosis and detects minimal residual disease in acute promyelocytic leukemia. *Proc. Natl Acad. Sci. USA*, **89**, 2694–2698.

MILLER WH, LEVINE K, DEBLASIO A, FRANKEL SR, DMITROVSKY E AND WARRELL RP. (1993). Detection of minimal residual disease in acute promyelocytic leukemia by a reverse transcription–polymerase chain reaction assay for the pml rar-alpha fusion messenger RNA. *Blood*, **82**, 1689–1694.

MIYAMURA K, TANIMOTO M, MORISHIMA Y, HORIBE K, YAMAMOTO K, AKATSUKA M, KODERA Y, KOJIMA S, MATSUYAMA K, HIRABAYASHI N, YAZAKI M, IMAI K, ONOZAWA Y, KANAMARU A, MIZUTANI S AND SAITO H. (1992). Detection of Philadelphia chromosome-positive acute lymphoblastic leukemia by polymerase chain reaction – possible eradication of minimal residual disease by marrow transplantation. *Blood*, **79**, 1366–1370.

MOERTEL CG, FLEMING TR, MACDONALD JS, HALLER DG, LAURIE JA, GOODMAN PJ, UNGERLEIDER JS, EMERSON WA, TORMEY DC, GLICK JH, VEEDER MH AND MAILIARD JA. (1990). Levamisole and fluorouracil for adjuvant therapy of resected colon carcinoma. *N. Engl. J. Med.*, **322**, 352–358.

MOLINO A, COLOMBATTI M, BONETTI F, ZARDINI M, PASINI F, PERINI A, PELOSI G, TRIDENTE G, VENERI D AND CETTO GL. (1991). A comparative analysis of three different techniques for the detection of breast cancer cells in bone marrow. *Cancer*, **67**, 1033–1036.

MOLL R, LOWE A, LAUFER J AND FRANKE WW. (1992). Cytokeratin 20 in human carcinomas. A new histodiagnostic marker detected by monoclonal antibodies. *Am. J. Pathol.*, **140**, 427–447.

MORENO JG, CROCE CM, FISCHER R, MONNE M, VIHKO P, MULHOLLAND SG AND GOMELLA LG. (1992). Detection of hematogenous micrometastasis in patients with prostate cancer. *Cancer Res.*, **52**, 6110–6112.

MORGAN GJ, HUGHES T, YANSSEN JWG, GOW J, GUO AP, GOLDMAN JM, WIEDEMANN LM AND BARTRAM CR. (1989). Polymerase chain reaction for detection of residual leukaemia. *Lancet*, **1**, 928–930.

MORRIS SW, KIRSTEIN MN, VALENTINE MB, DITTMER KG, SHAPIRO DN, SALTMAN DL AND LOOK, AT. (1994). Fusion of a kinase gene, Alk, to a nucleolar protein gene, Npm, in non-Hodgkin's lymphoma. *Science*, **263**, 1281–1284.

NAGAFUJI K, HARADA M, TAKAMATSU Y, ETO T, TESHIMA T, KAMURA T, OKAMURA T, HAYASHI S, AKASHI K AND MURAKAWA M. (1993). Evaluation of leukemic contamination in peripheral blood stem cell harvests by reverse transcriptase polymerase chain reaction. *Br. J. Haematol.*, **85**, 578–583.

NAGLE, RB. (1988). Intermediate filaments: a review of basic biology. *Am. J. Surg. Pathol.*, **12** (Suppl. 1), 4–16.

NAITO H, KUZUMAKI N, UCHINO J-I, KOBAYASHI R, SHIKANO T, ISHIKAWA Y AND MATSUMOTO S. (1991). Detection of tyrosine hydroxylase mRNA and minimal neuroblastoma cells by the reverse transcription–polymerase chain reaction. *Eur. J. Cancer*, **27**, 762–765.

NEALE GAM, MENARGUEZ J, KITCHINGMAN GR, FITZGERALD TJ, KOEHLER M, MIRRO JJ AND GOORHA RM. (1991). Detection of minimal residual disease in T-cell acute lymphoblastic leukemia using polymerase chain reaction predicts impending relapse. *Blood*, **78**, 739–744.

NEGRIN RS AND PESANDO J. (1994). Detection of tumor cells in purged bone marrow and peripheral blood mononuclear cells by polymerase chain reaction amplification of bcl-2 translocations. *J. Clin. Oncol.*, **12**, 1021–1027.

NIZET Y, VANDAELE S, LEWALLE P, VAERMAN JL, PHILIPPE M, VERMYLEN C, CORNU G, FERRANT A, MICHAUX JL AND MARTIAT P. (1993). Long-term follow-up of residual disease in acute lymphoblastic leukemia patients in complete remission using clonogeneic IgH probes and the polymerase chain reaction. *Blood*, **82**, 1618–1625.

NORRIS MD, GILBERT J, MARSHALL GM AND HABER M. (1994). Detection of minimal residual neuroblastoma by reverse transcription polymerase chain reaction. *Proc. Am. Assoc. Cancer Res.*, **35**, 1218.

OSBORNE M, WONG GY, ASINA S, OLD LJ, COTE RJ AND ROSEN PP. (1991). Sensitivity of immunocytochemical detection of breast cancer cells in human bone marrow. *Cancer Res.*, **51**, 2706–2709.

PELICCI PG, KNOWLES DM, MAGRATH I AND DALLA-FAVERA R. (1986). Chromosomal breakpoints and structural alterations of the c-Myc locus differ in endemic and sporadic forms of Burkitt lymphoma. *Proc. Natl Acad. Sci. USA*, **83**, 2984–2988.

PHILIP T, ARMITAGE JO, SPITZER G, CHAUVIN F, JAGANNATH S, CAHN J-Y, COLOMBAT P, GOLDSTONE AH, GORIN NC, FLESH M, LAPORTE J-P, MARANINCHI D, PICO J, BOSLY A, ANDERSON C, SCHOTS R, BIRON P, CABANILLAS F AND DICKE K. (1987). High-dose therapy and autologous bone marrow transplantation after failure of conventional chemotherapy in adults with intermediate-grade or high-grade non-Hodgkin's lymphoma. *N. Engl. J. Med.*, **316**, 1493–1498.

POTTER MN, STEWARD CG, MAITLAND NJ AND OAKHILL A. (1992). Detection of clonality in childhood B-lineage acute lymphoblastic leukemia by the polymerase chain reaction. *Leukemia*, **6**, 289–294.

PRICE CGA, MEERABUX J, MURTAGH S, COTTER FE, ROHATINER AZS, YOUNG BD AND LISTER TA. (1991*a*). The significance of circulating cells carrying t(14;18) in long remission from follicular lymphoma. *J. Clin. Oncol.*, **9**, 1527–1532.

PRICE CGA, ROHATINER AZS, STEWARD WP, DEAKIN DP, BAILEY N, NORTON A, BLACKLEDGE G, CROWTHER D AND LISTER TA. (1991*b*). Interferon-a2b in the treatment of follicular lymphoma: preliminary results of a trial in progress. *Ann. Oncol.*, **2** (Suppl. 2), 141–145.

PRIVERITERA E, KAMPS MP, HAYASHI Y, INABA T, SHAPIRO LH, RAIMONDI SC, BEHM F, HENDERSHOT L, CARROLL AJ, BALTIMORE D AND LOOK AT. (1992). Different molecular consequences of the 1 19 chromosomal translocation in childhood B-cell precursor acute lymphoblastic leukemia. *Blood*, **79**, 1781–1788.

RIETHMULLER G, SCHNEIDERGADICKE E, SCHLIMOK G, SCHMIEGEL W, RAAB R, HOFFKEN K, GRUBER R, PICHLMAIER H, HIRCHE H AND PICHLMAYR R. (1994). Randomized trial of monoclonal antibody for adjuvant therapy of resected Dukes C colorectal carcinoma. *Lancet*, **343**, 1177–1183.

ROTH MS, ANTIN JH AND GINSBURG D. (1989). Detection of Philadelphia chromosome-positive cells by the polymerase chain reaction following bone marrow transplant for chronic myelogenous leukemia. *Blood*, **77**, 874.

SAIKI RK, BUGAWAN TL, HORN GT, MULLIS KB AND ERLICH HA. (1986). Analysis of enzymatically amplified beta-globin and HLA-DQ alpha DNA with allele-specific oligonucleotide probes. *Nature*, **324**, 163–6.

SAVTCHENKO ES, SCHIFF TA, JIANG C-K, FREEDBURG IM AND BLUMENBURG M. (1988). Embryonic expression of the human 40-kD keratin: evidence from a processed pseudogene sequence. *Am. J. Hum. Genet.*, **43**, 630–637.

SAWYERS CL, TIMSON L, KAWASAKI ES, CLARK SS, WITTE ON AND CHAMPLIN R. (1990). Molecular relapse in chronic myelogenous leukemia patients after bone marrow transplantation detected by polymerase chain reaction. *Proc. Natl Acad. Sci. USA*, **87**, 563–567.

SCHLIMOK G, FUNKE I, HOLZMANN B, GOTTLINGER G, SCHMIDT G, HAUSER H, SWIERKOT S, WARNECKE HH, SCHNEIDER B, KOPROWSKI H AND RIETHMULLER G. (1987). Micrometastatic cancer cells in bone marrow: *in vitro* detection with anti-cytokeratin and *in vivo* labeling with anti-17-1A monoclonal antibodies. *Proc. Natl Acad. Sci. USA*, **84**, 8672–8676.

SCHOENFELD A, LUQMANI E, SMITH D, OREILLY S, SHOUSHA S, SINNET HD AND COOMBES RC. (1994). Detection of breast cancer micrometastases in axillary lymph-nodes by using polymerase chain reaction. *Cancer Res.*, **54**, 2986–2990.

SIDRANSKY D, TOKINO T, HAMILTON SR, KINZLER KW, LEVIN B, FROST P AND VOGELSTEIN B. (1992). Identification of ras oncogene mutations in the stool of patients with curable colorectal tumours. *Science*, **256**, 102–105.

SMITH B, SELBY P, SOUTHGATE J, PITTMAN K, BRADLEY C AND BLAIR GE. (1991). Detection of melanoma cells in peripheral blood by means of reverse transcriptase and polymerase chain reaction. *Lancet*, **338**, 1227–1229.

SOEKARMAN D, VONLINDERN M, VANDERPLAS DC, SELLERI L, BARTRAM CRI, MARTIAT P, CULLIGAN D, PADUA RA, HASPERVOOGT KP AND HAGEMEIJER A. (1992). Dek-can rearrangement in translocation (69)(p23 q34). *Leukemia*, **6**, 489–494.

STEWARD CG, GOULDEN NJ, KATZ F, BAINES D, MARTIN PG, LANGLANDS K, POTTER MN, CHESSELLS JM & OAKHILL A. (1994). A polymerase chain reaction study of the stability of Ig heavy-chain and T-cell receptor delta-gene rearrangements between presentation of childhood B-lineage acute lymphoblastic leukemia. *Blood*, **83**, 1355–1362.

SWIRSKY DM, LI YS, MATTHEWS JG, FLEMANS RJ, REESE JKH AND HAYHOE FGJ. (1984). 8;21 translocation in acute granulocytic leukaemia: cytological, cytochemical and clinical features. *Br. J. Haematol*, **56**, 119–213.

TADA M, OMATA M, KAWAI S, SAISHO H, OHTO M, SAIKI RK AND SNINSKY JJ. (1993). Detection of ras gene mutations in pancreatic juice and peripheral blood of patients with pancreatic adenocarcinoma. *Cancer Res.*, **53**, 2472–2474.

TAKVORIAN T, CANELLOS GP, RITZ J, FREEDMAN AS, ANDERSON KC, MAUCH P, TARBELL N, CORAL F, DALEY H, YEAP B, SCHLOSSMAN SF AND NADLER LM. (1987). Prolonged disease-free survival after autologous bone marrow transplantation in patients with non-Hodgkin's lymphoma with a poor prognosis. *N. Engl. J. Med.*, **316**, 1499–1505.

TKACHUK DC, KOHLER S AND CLEARY ML. (1992). Involvement of a homolog of *Drosophila trithorax* by 11q23 chromosomal translocations in acute leukemias. *Cell.*, **71**, 691–700.

TRAWEEK ST, LIU J AND BATTIFORA H. (1993). Keratin gene expression in nonepithelial tissues – detection with polymerase chain reaction. *Am. J. Pathol.*, **142**, 1111–1118.

UDOMSAKDI C, EAVES CJ, SWOLIN B, REID DS, BARNETT MJ AND EAVES AC. (1992). Rapid decline of chronic myeloid leukemic cells in long-term culture due to a defect at the leukemic stem cell level. *Proc. Natl Acad. Sci. USA*, **89**, 6192–6196.

VANRHEE F, LIN F, CULLIS JO, SPENCER A, CROSS NCP, CHASE A, GARICOCHEA B, BUNGEY J, BARRETT J AND GOLDMAN JM. (1994). Relapse of chronic myeloid leukemia after allogeneic bone marrow transplant – the case for giving donor leukocyte transfusions before the onset of hematologic relapse. *Blood*, **83**, 3377–3383.

VERES G, GIBBS RA, SCHERER SE AND CASKEY CT. (1987). The molecular basis of the sparse fur mutation. *Science*, **237**, 415–417.

VOGELSTEIN B, FEARON ER, HAMILTON SR, KERN SE, PREISINGER AC, LEPPERT M, NAKAMURA Y, WHITE R, SMITS AMM AND BOS JL. (1988). Genetic alterations during colorectal tumor development. *N. Engl. J. Med.*, **319**, 525–532.

VORMWALD-DOGAN V, NICKEL P, WILLEMSE M, THOME M AND TILGEN W. (1994). Prevalence of melanoma cells in peripheral blood of patients with malignant-melanoma stage I–IV – detection by polymerase chain reaction. *J. Invest. Dermatol.*, **103**, 405–405.

WILLIAMS ME, SWERDLOW SH, ROSENBERG CL AND ARNOLD A. (1992). Characterization of chromosome 11 translocation breakpoints at the bcl-1 and Prad-1 loci in centrocytic lymphoma. *Cancer Res.*, **52**, 5541s–5544s.

YAMADA M, WASSERMAN R, LANGE B, REICHARD BA, WOMER RB AND ROVERA G. (1990). Minimal residual disease in childhood B-lineage lymphoblastic leukemia: persistence of leukemic cells during the first 18 months of treatment. *N. Engl. J. Med.*, **323**, 448–455.

YAMAMOTO K, SETO M, IIDA S, KOMATSU H, KAMADA N, KOJIMA S, KODERA Y, NAKAZAWA S, SAITO H AND TAKAHASHI T. (1994). Reverse transcriptase polymerase chain reaction detects heterogeneous chimeric messenger RNAs in leukemias with 11q23 abnormalities. *Blood*, **83**, 2912–2921.

ZAKUT R, TOPALIAN SL, KAWAKAMI Y, MANCINI M, ELIYAHU S AND ROSENBERG SA. (1993). Differential expression of MAGE-1, -2, and -3 messenger RNA in transformed and normal human cell lines. *Cancer Res.*, **53**, 5–8.

ZOUBECK A, PFLEIDERER C, SALZER-KUNTSCHINK M, AMMAN G, WINDHAGER R, FINK FM, KOSCRELNIAK E, DELATTRE O, STREHL S, AMBROS PF, GADNER H AND KOVAR H. (1994). Variability of EWS chimaeric transcripts in Ewings tumors: a comparison of clinical and molecular data. *Br. J. Cancer*, **70**, 908–913.

ZUCMAN J, DELATTRE O, DESMAZE C, PLOUGASTEL B, JOUBERT I, MELOT T, PETER M, DEJONG P, ROULEAU G AND AURIAS A. (1992). Cloning and characterization of the Ewing's sarcoma and peripheral neuroepithelioma t(11 22) translocation breakpoints. *Genes Chrom. Cancer*, **5**, 271–277.